# Intrahepatic cholangiocarcinoma with a liver abscess due to hepatic actinomycosis

**DOI:** 10.1186/s40792-023-01625-8

**Published:** 2023-03-23

**Authors:** Tomoya Masuda, Kenta Kobashi, Ryoma Sugimoto, Hiroshi Ishii, Kensuke Tsunemitsu

**Affiliations:** Department of Surgery, Saiseikai Saijo Hospital, 269-1 Tsuitachi, Saijo-Shi, Ehime, 793-0027 Japan

**Keywords:** Liver abscess, Hepatic actinomycosis, Intrahepatic cholangiocarcinoma

## Abstract

**Background:**

Liver tumors with liver abscesses are unusual and rarely reported. In particular, studies of intrahepatic cholangiocarcinoma with liver abscesses due to hepatic actinomycosis have not been reported.

**Case presentation:**

A 73-year-old woman presented with swelling of the right hypochondrium. Computed tomography revealed a mass lesion that was continuous with the abdominal wall in the right lobe of the liver, suggesting a liver tumor invading the abdominal wall. A liver biopsy revealed intrahepatic cholangiocarcinoma with a liver abscess. The histopathological specimen contained bacterial masses of actinomycosis, and the cause of the liver abscess was determined to be hepatic actinomycosis. As a result of percutaneous drainage and antibiotic therapy, the part of the tumor attached to the abdominal wall disappeared; therefore, we assumed that most of the lesion was not cholangiocarcinoma but a liver abscess due to hepatic actinomycosis. Radical surgery for residual intrahepatic cholangiocarcinoma was performed after chemotherapy. Currently, the patient is alive without recurrence 2 years and 9 months after the operation.

**Conclusion:**

We encountered a difficult-to-diagnose case of intrahepatic cholangiocarcinoma with a liver abscess due to hepatic actinomycosis. A needle biopsy allowed early diagnosis and percutaneous drainage was an effective treatment.

## Background

Liver tumors with liver abscesses are rare, except as a rare complication of cancer therapies such as arterial embolization or percutaneous local therapy [1, 2]. Among liver tumors, intrahepatic cholangiocarcinoma initially accompanied by a liver abscess is even rarer than liver tumors with liver abscesses, and few studies have been reported. Prognosis is believed to be poor because of the difficulty of early diagnosis [3, 4]. We herein report a case of intrahepatic cholangiocarcinoma with a liver abscess. Liver abscess caused by hepatic actinomycosis—an extremely rare condition—has never been reported before.

## Case presentation

A 73-year-old woman presented with swelling of the right hypochondrium. The patient had a medical history of high blood pressure. Physical examination confirmed a subcutaneous mass in the right hypochondrium. No obvious tenderness or peritoneal signs were observed. Body temperature was 36.5 °C, blood pressure was 150/95 mmHg, and heart rate was 129 bpm. A blood test on admission (Table 1) showed an increase in inflammatory response with a white blood cell count of 19,200/mm^3^ and C-reactive protein of 8.3 mg/dL. All tumor markers (carcinoembryonic antigen 2.0 ng/mL, CA 19-9 9.9 U/mL, α-fetoprotein 3.0 ng/mL, and protein induced by vitamin K absence or antagonist 9 mAU/mL) were within reference ranges.

**Table 1:** Laboratory data

Hematology			Viral examination		
WBC	19,200	/μL	HBsAg	(–)	
RBC	374 × 10^4^	/μL	HCVAb	(–)	
Hb	11	g/dL			
Hct	34.4	%	Coagulation		
Plt	49 × 10^4^	/μL	PT	106	%
Biochemistry			Tumor marker		
TP	8.3	g/dL	CEA	2	ng/mL
Alb	2.9	g/dL	CA19-9	9.9	U/mL
AST	23	IU/L	AFP	3	ng/mL
ALT	12	IU/L	PIVKA-II	9	mAU/mL
T.Bil	0.58	mg/dL			
D.Bil	0.23	mg/dL			
ALP	289	IU/L			
γ-GTP	49	IU/L			
LDH	162	IU/L			
T-chol	135	mg/dL			
ChE	69	IU/L			
BUN	8.4	mg/dL			
Cre	0.66	mg/dL			
CRP	8.325	mg/dL			
HbA1c	5.2	%			
Na	135	mEq/L			
K	3.6	mEq/L			
Cl	97	mEq/L			
BS	139	mg/dL			

**Fig. 1:**
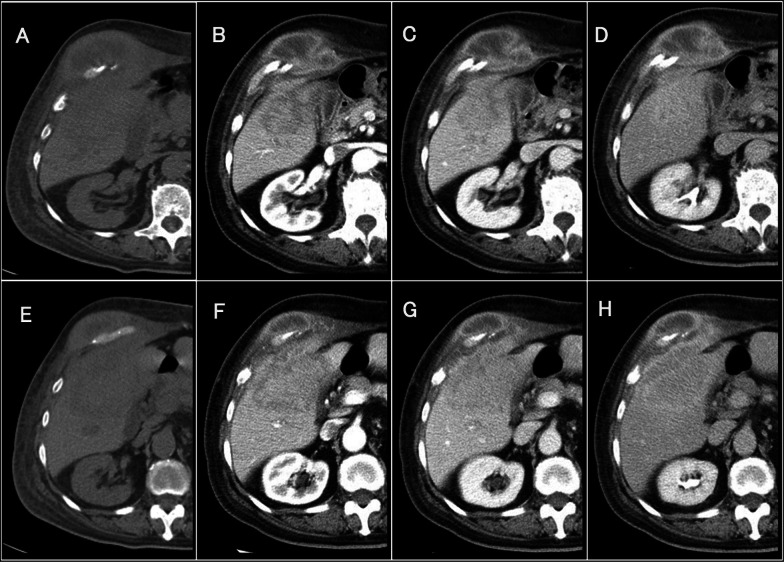
Abdominal computed tomography images before admission (**A**, **E** plain, **B**, **F** arterial phase, **C**, **G** portal phase, **D**, **H** equilibrium phase; **A**–**D** cranial site of the mass, and **E**–**H** caudal site of the mass)

**Fig. 2:**
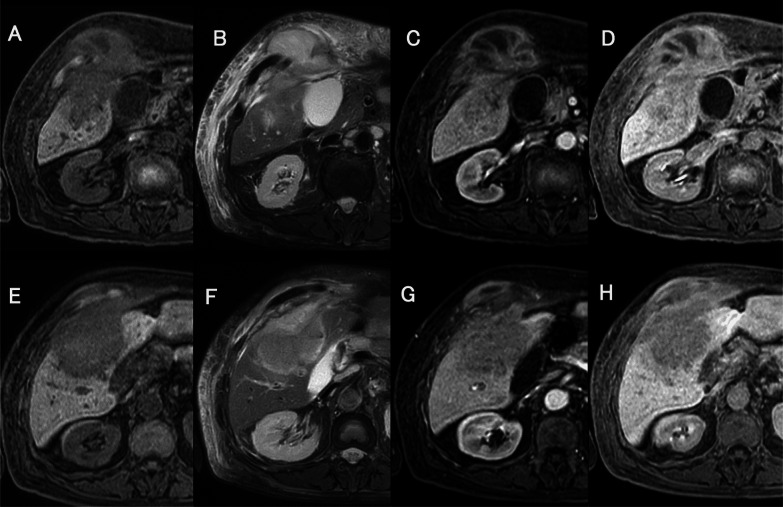
Enhanced magnetic resonance imaging before admission (**A**, **E** T1-weighted image, **B**, **F** T2-weighted image, **C**, **G** arterial phase, **D**, **H** hepatobiliary phase; **A**–**D** cranial site of the mass, and **E**–**H** caudal site of the mass)

A contrast-enhanced computer tomography (CT) scan showed a 68-mm mass lesion in the S5 of the right lobe of the liver. In the late phase, a strong enhancement effect was observed at the margin of the tumor. A 70-mm mass with poor contrast enhancement was found on the side of the abdominal wall. Because the mass was continuous with the liver, we suspected invasion of the abdominal wall by a hepatic malignant tumor such as intrahepatic cholangiocarcinoma (Fig. 1). Contrast-enhanced magnetic resonance imaging (MRI) showed a contrast-enhancing effect at the margin of the tumor in the right lobe S5 of the liver, and diffusion-weighted imaging showed high intensity inside the tumor. The finding was consistent with abdominal wall invasion by a malignant liver tumor (Fig. 2). On positron emission tomography/computed tomography, abnormal uptake was observed in a liver mass and a continuous abdominal wall mass (SUV max 14.0 [early phase], 19.1 [delayed phase]).

Based on the imaging findings, an invasion of the abdominal wall by a malignant hepatic tumor was suspected. However, a liver abscess could not be ruled out. A needle biopsy was performed for diagnostic purposes on the 14th day of hospitalization.

**Fig. 3:**
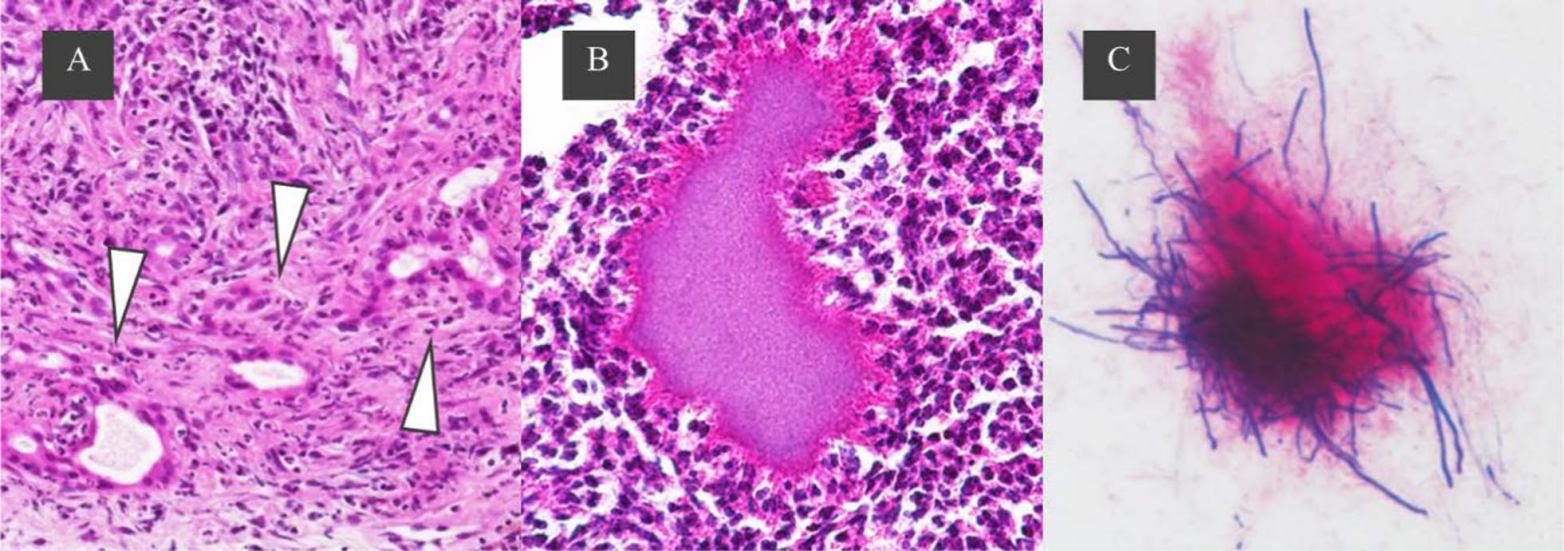
Needle biopsy findings. **A** Tubular adenocarcinoma with infiltrative growth of atypical ducts. **B** A typical sulfur granule (hematoxylin–eosin stain). **C** Tangled masses of branched and unbranched wavy bacterial filaments (Gram stain)

**Fig. 4:**
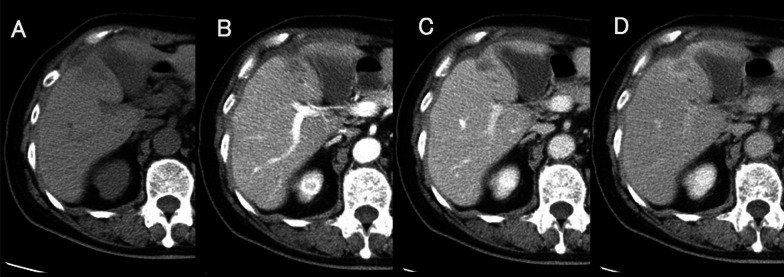
Abdominal computed tomography images after drainage and chemotherapy (**A** plain, **B** arterial phase, **C** portal phase, and **D** equilibrium phase)

A needle biopsy showed a white abscess in addition to liver tissue. Histopathological findings showed infiltrative growth of atypical ducts, and the patient was diagnosed with invasive cholangiocarcinoma with abscess formation (Fig. 3A). Percutaneous drainage was started the same day and the antibiotic tazobactam/piperacillin hydrate was administered for 14 days. A culture of the abscess detected *Fusobacterium* sp. but not actinomycetes. A hepatic abscess associated with hepatic actinomycosis was diagnosed because bacterial masses of actinomycetes (sulfur granules) were observed in the pathological histopathology (Fig. 3B). Tangled masses of branched and unbranched wavy bacterial filaments in Gram stain, which is characteristic of actinomycosis, were also observed (Fig. 3C). In this case, *Fusobacterium* was also detected in a culture of the liver abscess; however, from these pathological and bacteriological findings and the characteristic infiltrative CT image, we presume that actinomycosis was the main cause of the abscess. Given that the abdominal wall portion of the mass shrank by the 30th day of hospitalization, we assumed that the abdominal wall portion of the mass was an abscess rather than a tumor infiltration. To reduce the risk of forming a disseminated lesion associated with percutaneous needle biopsy, we performed neoadjuvant therapy. Four cycles of chemotherapy (gemcitabine and cisplatin) were administered to treat intrahepatic cholangiocarcinoma from the 56th day of hospitalization and CT imaging was performed again. A contrast-enhanced CT image on the 155th day of hospitalization showed that the hepatic mass in the late phase shrunk considerably to 27 mm in size, although the contrast effect remained (Fig. 4). Abnormal uptake in the mass disappeared on positron emission tomography/computed tomography. Contrast-enhanced MRI showed a contrast-enhancing effect on the mass in the early phase; however, no high-intensity regions were observed on diffusion-weighted imaging. Evaluating remnant intrahepatic cholangiocarcinoma using imaging alone was difficult. On the 198th day of hospitalization, a hepatectomy was performed after receiving informed consent from the patient.

Central bisegmentectomy of the liver, cholecystectomy, and lymphadenectomy in the hepatoduodenal ligament (No. 12) were performed. Only mild adhesions were observed between the liver and abdominal wall, and no disseminated lesions were seen in the abdominal wall or the intraperitoneal cavity. A rapid pathological diagnosis of the No. 12 lymph node and intra-abdominal nodule confirmed the absence of cancer metastasis.

**Fig. 5:**
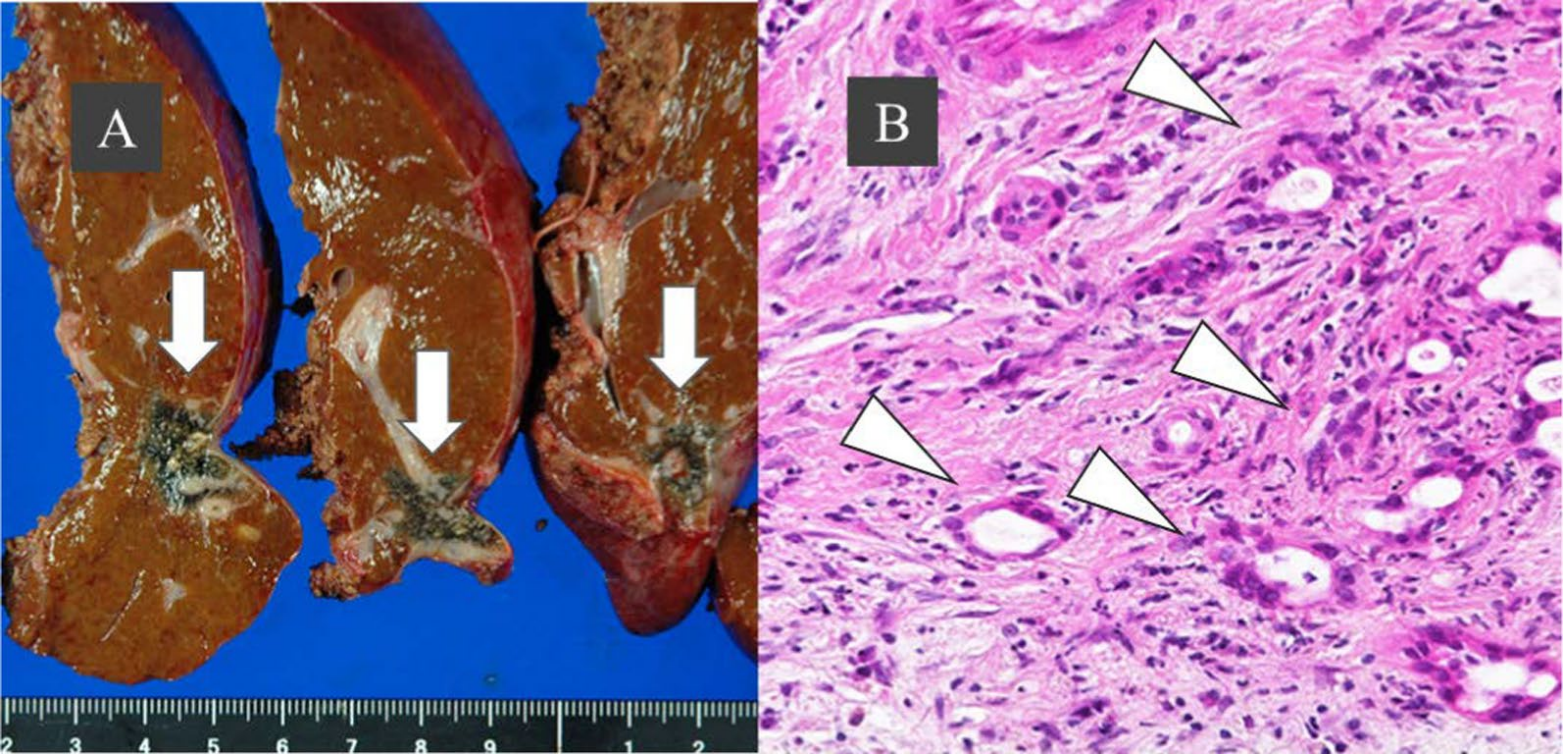
Pathological findings. Bile duct proliferation partially overlaps with obsolete inflammatory foci. Tumor size of 18 × 15 mm (arrow) excluding the scarred area. Highly differentiated intrahepatic cholangiocarcinoma with moderate atypia (arrowhead). **A** Macroscopic finding; **B** histological finding, hematoxylin–eosin stain

Histopathological findings showed an old inflammatory focus forming scars on the margin of the liver and a well-differentiated intrahepatic cholangiocarcinoma measuring 1.8 × 1.5 cm that partially overlapped with inflammatory foci. No invasion into the surrounding liver parenchyma, vascular invasion, extension to the serosal membrane, or invasion into the bile duct were observed (Fig. 5). No evidence of actinomycosis was observed in the resected liver tissue. The tumor was diagnosed as T1aN0M0 Stage IA in accordance with the Union for International Cancer Control tumor-node-metastasis classification stage (8th edition). The histopathological evaluation of the therapeutic effect was Grade IIa (i.e., 10% to 50% of tumor cells destroyed) by Evans classification and Score 2 (i.e., partial response: residual cancer with evident tumor regression, but more than single cells or rare small groups of cancer cells) by the College of American Pathologists.

The postoperative course was uneventful and the patient was discharged from hospital on the 214th day of hospitalization (or 16th postoperative day). The patient has been recurrence-free without adjuvant chemotherapy for 2 years and 9 months after surgery.

## Discussion

In this case, intrahepatic cholangiocarcinoma formed a mass resembling a liver abscess due to hepatic actinomycosis—an extremely rare condition that, to the best of our knowledge, has never been reported before. In general, malignant liver tumors with liver abscesses are misdiagnosed as refractory liver abscesses and cancer treatment is often delayed [2]. Without percutaneous liver biopsy in this case, treatment for intrahepatic cholangiocarcinoma would have been delayed. Using the tumor marker carbohydrate antigen 19-9 (CA-19-9) is useful to prevent overlooking the intrahepatic cholangiocarcinoma [3, 5]. However, despite the presence of intrahepatic cholangiocarcinoma in this case, CA 19-9 levels were within the reference interval and the marker was unhelpful for diagnosis.

In general, pyogenic liver abscesses are caused by enteric bacteria such as *Klebsiella pneumoniae* and *Escherichia coli* [6, 7]. Actinobacteria are unusual causes of liver abscesses.

Actinomycosis is a chronic pyogenic granulomatous disease mainly caused by *Actinomyces israelii*, an anaerobic, Gram-positive bacillus that normally resides in the oral cavity and gastrointestinal tract. Actinomycosis is observed in every organ; however, more than half of cases occur in the head and neck region and approximately 20% of cases occur in the abdomen. Hepatic actinomycosis accounts for approximately 5% of all actinomycoses and can cause liver abscesses [8–10]. Imaging findings of hepatic actinomycosis mimic those of malignancies, making diagnosis difficult. Both the histopathological detection rate of actinomycete (sulfur) granules and the detection rate of actinomycetes in bacterial culture are low [8, 9]; a definitive diagnosis cannot be made using imaging modalities alone. CT often shows single or multiple low-density shadows, which may be accompanied by enhancement; the border is often unclear in this type of image [8]. MRI shows a low-signal area on T1-weighted images and a high-signal area on T2-weighted images. These findings are similar to those for pyogenic liver abscess, intrahepatic cholangiocarcinoma, and inflammatory pseudotumor [8, 11]. Hepatic actinomycosis is difficult to differentiate from malignant tumors because of its infiltrative nature and its tendency to invade normal anatomic barriers [9, 12]. Although this case also agrees with general imaging findings of hepatic actinomycosis, diagnosing hepatic actinomycosis by imaging alone was impossible.

Hepatic actinomycosis is often misdiagnosed as hepatic malignancy and is diagnosed by postoperative histopathological examination [9]. In this case, actinomycosis was not detected by culture of the abscess, whereas a histopathological diagnosis of hepatic actinomycosis was confirmed by percutaneous liver biopsy. Actinomycosis is a chronic pyogenic granulomatous disease that can invade adjacent organs and form a mixed lesion with an abscess [8]. Actinomycosis can form fistulas in the skin [13]. In this case, invasion of the abdominal wall by a hepatic malignant tumor was suspected at first; however, the majority of the lesion was diagnosed as a liver abscess due to actinomycosis because the lesion was considerably reduced following percutaneous drainage and antibiotic treatment.

No consensus exists on the treatment of actinomycosis. Long-term high-dose administration of β-lactam antibiotics such as penicillin is recommended [8], whereas some reports argue that antibiotic therapy is unnecessary after the curative resection of lesions [14]. Appropriately treated hepatic actinomycosis has a favorable prognosis; however, delayed diagnosis leads to a fatal course [8, 9]. In this case, percutaneous drainage and administration of the antibiotic tazobactam/piperacillin hydrate resulted in a reduction in tumor size and the remission of actinomycosis within one month. Treatment for actinomycosis dramatically reduced tumor volume and we were able to minimize the hepatectomy for intrahepatic cholangiocarcinoma. Percutaneous drainage was useful for both diagnosis and treatment.

To the best of our knowledge, no study of intrahepatic cholangiocarcinoma with liver abscesses due to hepatic actinomycosis has been reported. Furthermore, this case was unusual and interesting because hepatic actinomycosis and intrahepatic cholangiocarcinoma were not two separate lesions but occurred within the same mass.

Immunosuppressive status is a known risk factor for invasive actinomycosis [10]. Because actinomycosis is an endogenous infectious disease, the time of infection and incubation period are unclear. However, in this case, we assume that immunosuppressive status due to being a cancer bearer is a risk for hepatic actinomycosis.

The percutaneous biopsy performed in this case may increase the risk of forming a disseminated lesion of the malignant tumor [3, 5]. In this case, intrahepatic cholangiocarcinoma was diagnosed by biopsy; thus, neoadjuvant chemotherapy was administered to prevent disseminated lesion formation. The patient has had an uneventful postoperative course without recurrence (including disseminated lesion formation) for 2 years and 9 months.

In this case, a percutaneous biopsy was useful for both diagnosis and treatment. Percutaneous biopsy enabled an early and accurate diagnosis and resulted in a good prognosis despite a complicated case of hepatic actinomycosis and intrahepatic cholangiocarcinoma.

## Conclusion

We encountered an extremely rare and difficult-to-diagnose case of intrahepatic cholangiocarcinoma with a liver abscess due to hepatic actinomycosis. A needle biopsy was useful in providing an early diagnosis and percutaneous drainage was an effective treatment.

## Data Availability

Not applicable.

## Abbreviations

CT: Computed tomography
CA19-9: Carbohydrate antigen 19-9
MRI: Magnetic resonance imaging
